# “It’s not only the bad side” - Experiences reported by health professionals working with women victims of sexual violence in a Brazilian university specialized outpatient service: A qualitative study

**DOI:** 10.1192/j.eurpsy.2022.2213

**Published:** 2022-09-01

**Authors:** E. Turato, J. Cavalcante, F. Silva, L. Guerra, R. Azevedo

**Affiliations:** State University of Campinas, Laboratory Of Clinical-qualitative Research, Campinas, Brazil

**Keywords:** mental health care, health professionals, Qualitative research, sexual violence

## Abstract

**Introduction:**

Health Psychology is a scientific branch that studies interpersonal relationships in the field of emotions and behavior in clinical settings. Violence against women is a gender-based action that alarmingly affects the population, with sexual violence (SV) being one of its main phenomena. The complexity of the care offered to SV patients by clinical professionals impacts themselves, affecting their personal lives and the quality of their work.

**Objectives:**

To explore symbolic emotional meanings attributed by health professionals to care and follow-up of women victims of SV in state service of reference of the Unified Health System.

**Methods:**

Clinical-Qualitative design was used to guide semi-directed interviews with open-ended questions in-depth. Clinical-Qualitative Content Analysis was employed to treat data. Five participants make up the multi-professional team at the Hospital of the Woman of the State University of Campinas. Theoretical framework chosen to interpret categories was Balintian Medical Psychology.

**Results:**

Three categories were selected for this presentation: The human anguishes as the main challenge and handling of working with sexual violence; “To see things progressing”: to the patient and together with the team, a facilitator of the work; and “I try to leave it on the three’s leaves”: the difficult attempt to separate work from personal life.

**Conclusions:**

Taking care of SV is a very emotionally demanding task. Working with the team and see expected outcomes help clinical professionals deal with negative feelings, avoiding, for example, compensatory traumas. New research about social-cultural impacts of working with SV is important to develop institutional approaches of coping for health teams.

**Disclosure:**

No significant relationships.

